# Diverse functional elements in RNA predicted transcriptome-wide by orthogonal RNA structure probing

**DOI:** 10.1093/nar/gkab885

**Published:** 2021-10-11

**Authors:** Dalen Chan, Chao Feng, Whitney E England, Dana Wyman, Ryan A Flynn, Xiuye Wang, Yongsheng Shi, Ali Mortazavi, Robert C Spitale

**Affiliations:** Department of Pharmaceutical Sciences, University of California, Irvine. Irvine, CA 92697, USA; Department of Pharmaceutical Sciences, University of California, Irvine. Irvine, CA 92697, USA; Department of Pharmaceutical Sciences, University of California, Irvine. Irvine, CA 92697, USA; Department of Developmental and Cellular Biology, University of California, Irvine. Irvine, CA 92697, USA; Stem Cell Program, Boston Children’s Hospital, Boston, MA, USA and Department of Stem Cell and Regenerative Biology, Harvard University, Cambridge, MA, USA; Department Microbiology and Molecular Genetics, University of California, Irvine. Irvine, CA 92697, USA; Department Microbiology and Molecular Genetics, University of California, Irvine. Irvine, CA 92697, USA; Department of Developmental and Cellular Biology, University of California, Irvine. Irvine, CA 92697, USA; Department of Pharmaceutical Sciences, University of California, Irvine. Irvine, CA 92697, USA; Department of Chemistry, University of California, Irvine. Irvine, CA 92697, USA

## Abstract

RNA molecules can fold into complex structures and interact with trans-acting factors to control their biology. Recent methods have been focused on developing novel tools to measure RNA structure transcriptome-wide, but their utility to study and predict RNA-protein interactions or RNA processing has been limited thus far. Here, we extend these studies with the first transcriptome-wide mapping method for cataloging RNA solvent accessibility, icLASER. By combining solvent accessibility (icLASER) with RNA flexibility (icSHAPE) data, we efficiently predict RNA-protein interactions transcriptome-wide and catalog RNA polyadenylation sites by RNA structure alone. These studies showcase the power of designing novel chemical approaches to studying RNA biology. Further, our study exemplifies merging complementary methods to measure RNA structure inside cells and its utility for predicting transcriptome-wide interactions that are critical for control of and regulation by RNA structure. We envision such approaches can be applied to studying different cell types or cells under varying conditions, using RNA structure and footprinting to characterize cellular interactions and processing involving RNA.

## INTRODUCTION

Precise structural conformations are a hallmark of functional nucleic acid polymers in cells. For example, the genome is arranged in a compact three-dimensional structure, whose dynamics and solvent accessibility are critical for the control of gene expression (1). Methods to measure these structural properties of the genome are now quite mature and can be done genome-wide to understand the interactions between DNA and proteins, and even predict RNA expression outcomes (2). Recently, there has been an emergence of approaches to measure transcriptome-wide RNA structure in cells (3), but these efforts still lag behind in their utility and impact when compared to the biological significance of DNA-centric measurements discussed above.

The ability of RNA molecules to fold into complex two- and three-dimensional structures is critical for the many biological functions they perform (4,5). Currently, the majority of reagents used for structure probing can identify base-paired residues within RNA. For example, adenosine and cytosine residues not involved in Watson-Crick-Franklin pairing can be alkylated by dimethylsulfate (6). Similarly, glyoxals have recently been observed to react with guanosine residues at N-1 and exocyclic N-2 position (7). Selective hydroxyl acylation analyzed by primer extension, or SHAPE, approximates 2′-hydroxyl flexibilities (8,9). Together, the wide adoption and use of these chemicals demonstrate the power of regent design to understand and relate chemical reactivity to RNA structure.

**Figure 1.**
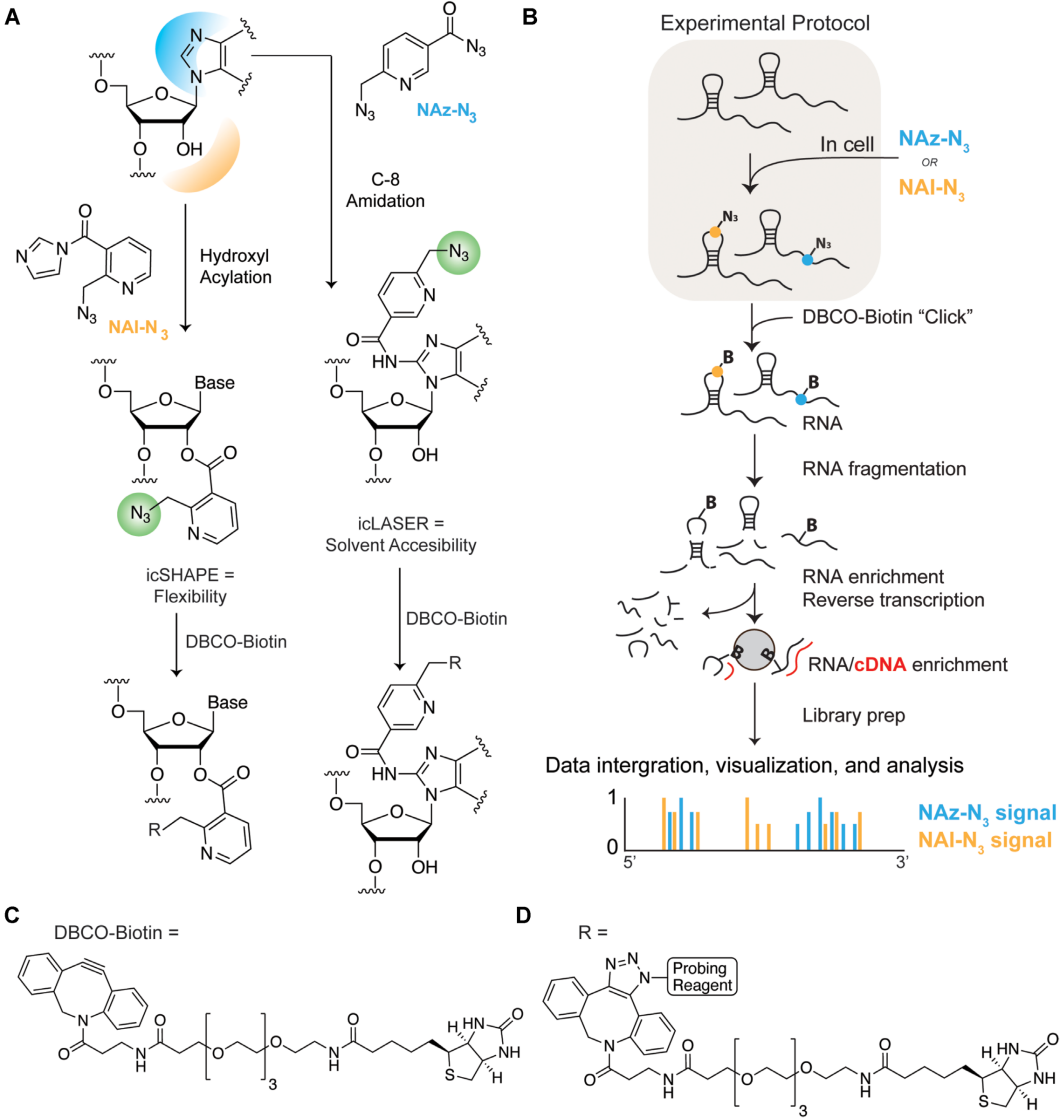
Schematic for icSHAPE and icLASER. (**A**) Chemical protocol for icSHAPE (10) and icLASER. (**B**) Protocol for library preparation for icSHAPE and icLASER, which could be integrated in the same protocol method for RNA structure probing, transcriptome-wide. (**C**) Chemical structure of dibenzylcyclooctyne biotin (DBCO biotin) used for copper-free ‘click’ reaction with azido-modified structure probes. (**D**) Chemical structure of the final product between azido-modified structure probes and DBCO biotin.

Transferring conventional chemical probes from one-RNA-at-a-time measurements to transcriptome-wide scale has proven to be a formidable challenge. Bifunctional RNA structure probing reagents that enable reactivity with RNA and enrichment of reaction sites has been demonstrated to dramatically increase signal-to-noise ratios of structural information per read arising from chemical adducts (10,11). We previously developed icSHAPE, which features a bifunctional chemical probe to measure nucleobase flexibility through RNA hydroxyl acylation transcriptome-wide (10) (Figure 1A).

Solvent accessibility, an orthogonal property of RNA that is powerful for characterizing RNA-protein interactions (12), has traditionally been more difficult to probe inside cells, requiring the use of a synchrotron radiation light source (13). We recently described the development of LASER, or Light Activated Structural Examination of RNA (14). LASER relies on the facile light activation of a nicotinoyl azide to a nitrenium ion, which undergoes electrophilic aromatic substitution with electron rich purine residues at their C8 nucleobase position. While this initial work demonstrated the utility of LASER to read out solvent accessibility, it still remained a critical challenge to extend these efforts to a transcriptome-wide scale and evaluate this approach to understand RNA structure and function more broadly. Herein we describe our efforts in expanding the toolbox of RNA structure probing reagents, integrating the reactivity of multiple probes together, and taking the important step of demonstrating their utility to understand RNA functional elements including RNA processing and RNA-protein interactions sites.

## MATERIALS AND METHODS

All methods pertaining to the synthesis of all probes, as well as library construction for structure probing and polyadenylation sequencing, are presented in the Supplementary Information.

### Primers used for reverse transcription

SAM-I (5′, ATTTAGGTGACACTATAGTT, 3′)

18s Primer 1 (5′, CCAATTACAGGGCCTCGAAA, 3′)

### SAM-I construct (control RNA for analysis of NAz-N_3_ structure probing)

A 94 nucleotide construct consisting of the sequence for the SAM-I riboswitch from the metF-metH2 operon of *T. tencongensis* was designed into a plasmid with IDT. The plasmid encoding the SAM-I sequence was transformed into a One Shot Top 10 chemically competent cells (ThermoFisher) and plated on lysogeny broth (LB, ThermoFischer) supplemented with 100 mg/ml ampicillin (VWR) agar plates. A single colony was selected in a 3 ml culture and grown overnight. The resulting plasmid was isolated using a QIAprep Miniprep kit (Qiagen). Transcription template was prepared by PCR in 50 μl volumes using primers directed against the T7 promoter (5′, TAATACGACTCACTATAGGG, 3′) and an adaptor sequence for reverse transcription (5′, ATTTAGGTGACACTATAGTT, 3′) with Phusion High-Fidelity PCR master mix (NEB). The mixture was treated with 1 μl of DPN1 (NEB) restriction enzyme and incubated at 37°C for 30 min. The resulting PCR product was purified using a QIAquick PCR purification kit (Qiagen). *In vitro* transcription of SAM-I RNA was performed in 20 μl reactions using 800 ng of template SAM-I DNA with a T7 Ribomax transcription kit (Promega). Samples were treated with 1 μl of RQ1 DNAse (Promega) at 37°C for 30 min. The resulting solution was brought up to 100 μl and precipitated with 10 μl of 3 M NaOAc (pH 5.2), 1 μl glycoblue (ThermoFisher), and 300 μl ethanol. The precipitated RNA stored at −80°C for 30 min, centrifuged at 15 000 rpm for 15 minutes, and resuspended in 50 μl of RNase-free water. An aliquot of SAM-I was run on a denaturing PAGE gel (15% polyacrylamide, 0.5× TBE, 7 M urea) alongside a 100 bp RNA ladder (Invitrogen). The band of interest was visualized with 1× SYBR-gold stain (ThermoFisher) in water for 15 min. Resulting concentrations of RNA was quantified by integrating intensity of the ladder with the RNA band of interest.

### ^32^P End labeling for reverse transcription

Primer DNA was 5′ end labeled in 10 μl reactions in a T4 PNK mix (1 μl 10× T4PNK reaction buffer, 2 μl 100 mM DNA primer, 5 μl nuclease free water, 1 μl γ-^32^P-ATP, 1 μl T4 PNK, NEB). The reaction was allowed to proceed for 2 h at 37°C. Reactions were stopped by the addition of 5 μl of Gel Loading Buffer II. The reaction was loaded onto a 15% denaturing PAGE gel. The band of interest was visualized by a phosphorimager (Typhoon, GE healthcare). The resulting band was excised and eluted overnight in 400 μl of 300 mM KCl. Resulting solution was EtOH precipitated and dissolved to 8000 counts per minute (cpm)/μl for further use in reverse transcription.

### Modification of RNA *in vitro*

5 μg total RNA (isolated from HeLa cells) or 10 pmol of *in vitro* transcribed RNA in 6 μl metal-free water was heated for 2 min at 95°C. The RNA was then flash cooled on ice for 1 min, and brought to room temperature. 3 μl of 3× RNA folding buffer (333 mM HEPES, pH 8.0, 20 mM MgCl_2_ and 333 mM NaCl) was added, and the RNA was allowed to equilibrate at 37°C for 5 min. To this mixture 1 μl of 3 M NAz, 1 M NAz-N_3_ in DMSO (+) or DMSO (−) was added. Reactions were then exposed to 20 W lamp (Zilla Desert UVB 50) UV light for 3 min for NAz, and 10 minutes for NAz-N_3_. Reactions were brought up to 200 μl water, and extracted once with 200 μl acid phenol/chloroform/isoamyl alcohol (pH 4.5, Ambion), and washed twice with 200 μl chloroform (Sigma). Samples were precipitated by adding 20 μl 3 M NaOAc (pH 5.2), 1 μl glycoblue (20 μg/μl) and 600 μl EtOH. Pellets were washed twice with 70% cold ethanol and resuspended in 5 μl nuclease free water.

### Copper free click chemistry of modified RNA

In a reaction volume of 50 μl, modified RNA (10 pmol) was incubated with 5 μl dibenzocyclooctyne–PEG4–biotin (DBCO-Biotin, 10 mM, Sigma) for 1 h at 37°C in 1× PBS. Reactions were brought up to 200 μl and extracted once with 200 μl acid phenol/chloroform/isoamyl alcohol (pH 4.5), and washed twice with 200 μl chloroform. Samples were precipitated by adding 20 μl of 3 M NaOAc (pH 5.2), 1 μl glycoblue (20 μg/μl) and 600 μl EtOH. Pellets were washed twice with 70% cold ethanol and resuspended in 2 μl nuclease free water for dot blot analysis, or 10 μl nuclease free water for streptavidin enrichment assays.

### Dot blot analysis of enriched modified RNA

Hybond N + membranes (GE) were pre-incubated in 10× SSC. Precipitated biotinylated total RNA was dissolved in 2 μl of RNase free water. RNA was loaded onto the Hybond membrane and crosslinked using 254 nm ultraviolet light. The membrane was incubated with blocking solution (120 mM NaCl, 16 mM Na_2_HPO_4_, 8 mM NaH_2_PO_4_, 170 mM SDS) for 30 min. To the membrane was added 1 μl Pierce high sensitivity streptavidin-HRP(ThermoFisher) in blocking solution. The membrane was washed twice with wash buffer A (1:10 blocking solution) for 30 min, and twice with wash buffer B (100 mM Tris pH 9.5, 100 mM NaCl, 20 mM MgCl_2_) for 5 min. Membrane was incubated with Pierce western blotting substrate and visualized on the ChemiDoc (Biorad) under chemiluminesence hi sensitivity. To visualize RNA, the membrane was stained with a methylene blue solution (0.2% w/v methylene blue, 0.4 M sodium acetate).

### Modification of RNA in cells

HeLa cells were grown in DMEM (4.5 g/l glucose & l-glutamine [–] sodium pyruvate, Thermofisher) culture medium supplemented with 10% FBS (SAFC) and 1% penicillin streptomycin (Life technologies). K562 cells were grown in RPMI 1640 supplemented with 10% FBS, and 1% penicillin streptomycin. Cells were washed three times with Dulbecco's phosphate-buffered saline (DPBS, Genesee) and centrifuged at 1000 RPM for 5 min. Cells (∼3–6 × 10^7^) were resuspended in 45 μl DPBS. 5 μl DMSO (−), 10% final concentration, 5 μl 3 M NAz or 1 M NAz-N_3_ in DMSO (+) was added to the desired final concentration. Cell suspensions were subjected to UV light for 5 min using NAz and 10 minutes with NAz-N_3_. Cells were pelleted by centrifugation at 1000 RPM for 5 min and resuspended in 1 ml Trizol Reagent (ThermoFisher). RNA was harvested using Trizol Reagent following the manufacturer's instructions. 500 μl of aqueous phase was then precipitated with 500 μl isopropyl alcohol at room temperature for 10 min. Samples were centrifugated at 15 000 RPM at 4°C and washed twice with cold 70% EtOH.

### Enrichment of modified RNA

In 700 μl reaction volume, 50 pmol of biotinylated RNA was added with 50 μl of prewashed Dynabeads MyOne C1 beads (ThermoFisher). The solution was then mixed at room temperature for 1 h. The beads were collected on a magnetic plate and flowthrough was saved. The beads were then washed three times with 700 μl of Biotin Wash buffer (10 mM Tris–HCl, pH 7.0, 1 mM EDTA, 4 M NaCl, 0.2% Tween). The first wash was saved and combined with the flowthrough for further analysis. Samples were later washed twice with RNase-free water. NAz-N_3_ adducts underwent harsher wash conditions and were incubated twice with 700 μl Biotin wash buffer for 5 min along with two washes with RNase-free water at 70°C. Samples were eluted twice with 44 μl formamide, 1 μl of 0.5 M EDTA, and 5 μl of 50 mM of free D-Biotin at 95°C for 5 min. Eluted samples were diluted with 600 μl RNase-free water. All samples were purified using RNA Clean and Concentrator Kit (Zymo). Samples were eluted in 6 μl of RNase-free water and used for subsequent reverse transcription.

### Reverse transcription of modified RNA (in vitro and in vivo) for manual footprinting

^32^P-end-labeled DNA primers were annealed to modified RNA by incubating at 95°C for two minutes, then 25°C for 2 min, and 4°C for 2 min. To the reaction, 1 μl of 5× First strand buffer, 0.5 μl nuclease free water, 0.5 μl 100 mM DTT, and 0.5 μl 10 mM dNTP’s were added. The reaction was preincubated at 52°C for 1 min, then 1 μl superscript III (ThermoFisher) was added. Extensions were performed for 15 min. To the reaction, 1 μl 4 M sodium hydroxide was added and allowed to react for 5 min at 95°C. The resulting complementary DNA (cDNA) was snap cooled on ice, and ethanol precipitated according to above procedures. Purified cDNA was resuspended in 2 μl of nuclease-free water and 2 μl of Gel Loading Buffer II. cDNA products were resolved on 10% denaturing polyacrylamide gel, and visualized by a gel imager (Typhoon, GE healthcare).

### icLASER and icSHAPE sequencing preparation

Cells were treated with NAI-N_3_, NAz-N_3_ or DMSO, RNA extracted, and coupled to DBCO-biotin, as described previously (15). All samples were processed in the same way to first enrich for the poly-A fraction and then construct deep sequencing libraries. Poly-A enrichment was achieved using the Poly(A)Purist MAG Kit (Thermo Fisher Scientific). Typically, 200 μg total RNA was used as input. The manufacturer's protocol was followed, and performed twice on each sample, after which enriched polyA-tailed transcripts were desalted with the Zymo RNA clean and concentrator (Zymo Research) as per the manufacturer's protocol.

#### RNA fragmentation, end repair, ligation and adaptor clean up

RNA samples, typically 100 ng for DMSO and 1000 ng for NAI-N_3_ or NAz-N_3_ modified RNA, were first dried with a lyophilizer (Labconco) and brought back up 9 μl water. RNA was fragmented by adding 1 μl of Fragmentation Reagent (Ambion) at 70°C for 30 s and then stopped by adding 1 μl of RNA Fragmentation Stop Solution (Ambion) and moved to ice. RNA was desalted with a Zymo RNA clean and concentrator as above and again dried with a lyophilizer. End repair occurred by adding 5 μl of water, 2 μl of 5x PNK buffer (350 mM Tris–HCl pH 6.5, 50 mM MgCl_2_, 25 mM DTT), 0.5 μl SUPERaseIn (Thermo Fisher Scientific), and 1.5 μl of T4 PNK (NEB) and 1.5 μl Fast AP (Thermo Fisher Scientific). These reactions were incubated at 37°C for 45 min. After end-repair, 1 μl of 2 μM 3′ pre-adenylated adaptor (3′biotin linker for DMSO or 3′dideoxycytosine linker for NAI-N_3_ or NAz-N_3_), 1 μl of 10× T4 RNA Ligase I buffer (NEB), 1 μl T4 RNA Ligase, High Concentration (NEB), 1 μl of 100 mM DTT and 6 μl of 50% PEG8000 were added. Ligation reactions were incubated at 25°C for 6 h. Excess, unligated adaptors were destroyed enzymatically by adding 3 μl of 10× NEB Buffer 2 (NEB), 2 μl Rec Jf (NEB), 1 μl 5′ deadenylase (NEB), 4 μl of water and incubate at 37°C for 1 h. Ligated RNA products were desalted and removed of enzymes with the Zymo RNA clean and concentrator.

#### Biotin enrichment, cDNA synthesis, and cDNA circularization

Biotinylated RNAs (via 3′-biotin linker for DMSO libraries or NAI-N_3_, NAz-N_3_ modifications to the RNA) were captured by addition of 5 μl of MyOne Streptavidin C1 Dynabeads (ThermoFisher Scientific) that had been rinsed and suspended in 50 μl of Biotin-IP buffer (100 mM Tris, pH 7.5, 1 M NaCl, 1 mM EDTA, 0.1% Tween), and rotation for 45 min at 25°C. Non-biotinylated RNA was washed away with five additional washes of Biotin-IP buffer at 25°C and then excess detergent and salt were reduced by two bead rinses in NT2 buffer (50 mM Tris–HCl pH 7.5, 150 mM NaCl, 1 mM MgCl_2_, 0.05% NP-40). Beads were resuspended in 12 μl water 1 μl of 3 μM RT primer (16) and 1 μl of 10 mM dNTPs and heated to 70°C for 5 min then rapidly cooled to 4°C. cDNA Master Mix (4 μl 5× Super Script IV (SSIV) Buffer, 1 μl 100 mM DTT, 1 μl SSIV, 6 μl total) was added to the annealed RNA and incubated for 30 min at 55°C. Beads were placed on a 96-well magnet and washed sequentially twice with 100 μl of Biotin-IP buffer and 100 μl ice-cold 1× PBS. Beads were resuspended in 6 μl of pure water and headed to 95°C for 5 min, placed immediately on a 96-well magnet and eluted cDNA was transferred to a fresh PCR tube. This process was repeated once. Next 5 μl of circularization reaction buffer (3.3 μl water, 1.5 μl 10x Circligase-II buffer and 0.5 μl of Circligase-II (Epicentre)). cDNA was circularized for 2.5 h at 60°C. cDNA was purified with 30 μl of AMPure XP beads (Beckman Coulter) and 75 μl of isopropanol. Samples were incubated for 20 min at 25°C, washed twice with 100 μl 80% ethanol, air dried for 5 min, and eluted in 16 μl of water. Elution took place at 95°C for 3 min and immediately transferred to a 96-well magnet.

#### PCR amplification and library size selection

Eluted cDNA was transferred to a new PCR tube containing 15 μl of 2X Phusion HF-PCR Master Mix (NEB), 0.5 μl of 30 μM P3/P6 PCR1 oligo mix (16) and 0.5 μl of 15× SYBR Green I (ThermoFisher Scientific). Real-time quantitative PCR was preformed: 98°C 2 min, 15 cycles of 98°C 15 s, 65°C 30 s, 72°C, 30 s, with data acquisition set to the 72°C extension. PCR1 reactions were cleaned up by adding 54 μl of AMPure XP beads and incubation for 20 min. Beads were washed once with 80% ethanol, dried for 5 min, and eluted in 15 μl of water. Libraries were further purified using Native PAGE separation, selecting PCR products corresponding to cDNA insert of 20 bp or larger. Library DNA was eluted from cut sections of acrylamide by crushing the gel slice and incubating it in 300 μl of Crush Soak Buffer (500 mM NaCl, 1 mM EDTA, 10 mM Tris pH 7.5, 0.1% SDS) overnight at 55°C. Gel pieces were subsequently removed over a Spin-X column (Corning) and PCR products desalted with a DNA clean and concentrator column (Zymo research), eluting the products in 20 μl water. Illumina flow cell adaptors were added by adding 20 μl 2X Phusion HF-PCR Master Mix and 0.4 μl P3solexa/P6solexa oligo mix (16) and amplified: 98°C 2 min, 3 cycles of 98°C 15 s, 65°C 30 s, 72°C, 30 s. Final libraries were purified by addition of 48 μl of AMPure XP beads and incubation for 5 min. Beads were washed twice with 70% ethanol, dried for 5 min, and eluted in 20 μl of water. 2 μl of libraries were quantitated by HS-DNA Bioanalyzer (Agilent). Samples were deep sequenced on the Illumina NextSeq machine: single-end, no index, high-output, 75-bp cycle run.

### Generation of PolyA sequencing libraries

10 μg total RNA extracted with Trizol (Ambion) was fragmented with fragmentation reagent (Ambion) at 70°C for 10 min followed by precipitation with ethanol. Reverse transcription was performed with PASSEQ7-2 RT oligo:

- [phos]NNNNAGATCGGAAGAGCGTCGTGTTCGGATCCATTAGGATCCGAGACGTGTGCTCTTCCGATCTTTTTTTTTTTTTTTTTTTTVN

and Superscript III (Invitrogen). cDNA was recovered by ethanol precipitation and 120–200 nucleotides of cDNA was gel-purified from 8% urea–PAGE. Recovered cDNA was circularized with Circligase™ II (Epicentre) at 60°C overnight. Buffer E (Promega) was added to the cDNA and heated at 95°C for 2 min, and then cool to 37°C slowly. Circularized cDNA was linearized by BamH I (Promega). cDNA was collected by centrifugation after ethanol precipitation. PCR was carried out with primers PE1.0 and PE2.0 containing index (Illumina). Around 200 bp of PCR products was gel-purified and submitted for sequencing (single read 100 nucleotides).

### Data analysis for icSHAPE and icLASER

Raw sequence reads were quality-checked using FastQC (17) and demultiplexed. Reads were aligned to the ENSEMBL Release 88 GRCh38 transcriptome (18)and per-position enrichment scores were calculated using the icSHAPE pipeline (15). Default parameters were used with the exception of the final filtering step, where minimum values for hit coverage and background base density were removed (-T 0 -t 0).

Publicly available eCLIP peak data for five RNA binding proteins in K562 cells was downloaded from the ENCODE project (19). Known 5 bp binding motifs (BiorXiv https://doi.org/10.1101/179648) for each protein were located in each peak; peaks lacking the motif were discarded. icLASER enrichment scores were extracted for each motif position, plus five basepairs up- and downstream from the motif. Negative control sites were identified as occurrences of the same 5 bp motif that fall outside of eCLIP peaks. As with binding sites, icLASER enrichment scores were extracted for a 15 bp range centered on the motif. A number of negative sites equal to the number of positive sites was selected randomly from the pool of possible negatives.

icLASER analysis at poly-A signal sequences was conducted similarly; however, Ensembl-annotated polyadenylation signal sequences were used to identify positive sites, and enrichment scores were retrieved for a range of 20 bp up- and downstream of the 6 bp polyadenylation signal motif.

### SVM analysis of icSHAPE/icLASER and RNA-binding proteins

SVMs were implemented using libSVM (15). Both training and test data were scaled to a 0–1 scale and SVM parameters were selected using 5-fold cross-validation. For each motif, both *in vitro* and *in vivo* data sets were divided in half at random; half for the data was used to train the SVM, while the remaining half was used to test its predictions. icLASER scores at each position in the 15bp range surrounding the motif of interest were input as features. Ranges containing null values for icSHAPE or icLASER enrichment scores were discarded.

### SVM analysis of icSHAPE/icLASER and polyadenylation sites

SVM analysis was conducted as with RNA-binding proteins. icSHAPE or icLASER scores for a 46 bp range centered on the polyadenylation signal sequence were used as features.

## RESULTS

### Development of a bifunctional LASER probe

We designed a bifunctional LASER probe to measure transcriptome-wide purine C8 solvent accessibility (Figure 1A, *in vivo* click LASER, icLASER). Towards the goal of integrating multiple, orthogonal measurements of RNA structure, we set out to directly compare icSHAPE (hydroxyl acylation; flexibility) and icLASER (solvent accessibility) and use these measurements to predict a verity of functional element with RNAs across the transcriptome (Figure 1B). The design of these reagents employ an alkyl azide functional group for enrichment, which we predicted would not be light-sensitive and therefore can be used similarly to NAI-N_3_ as in icSHAPE to ligate biotin through copper-free ‘click’ reactions with dibenzylcyclooctyne-conjugated to biotin (DBCO-biotin) (Figure 1C and D)

**Figure 2.**
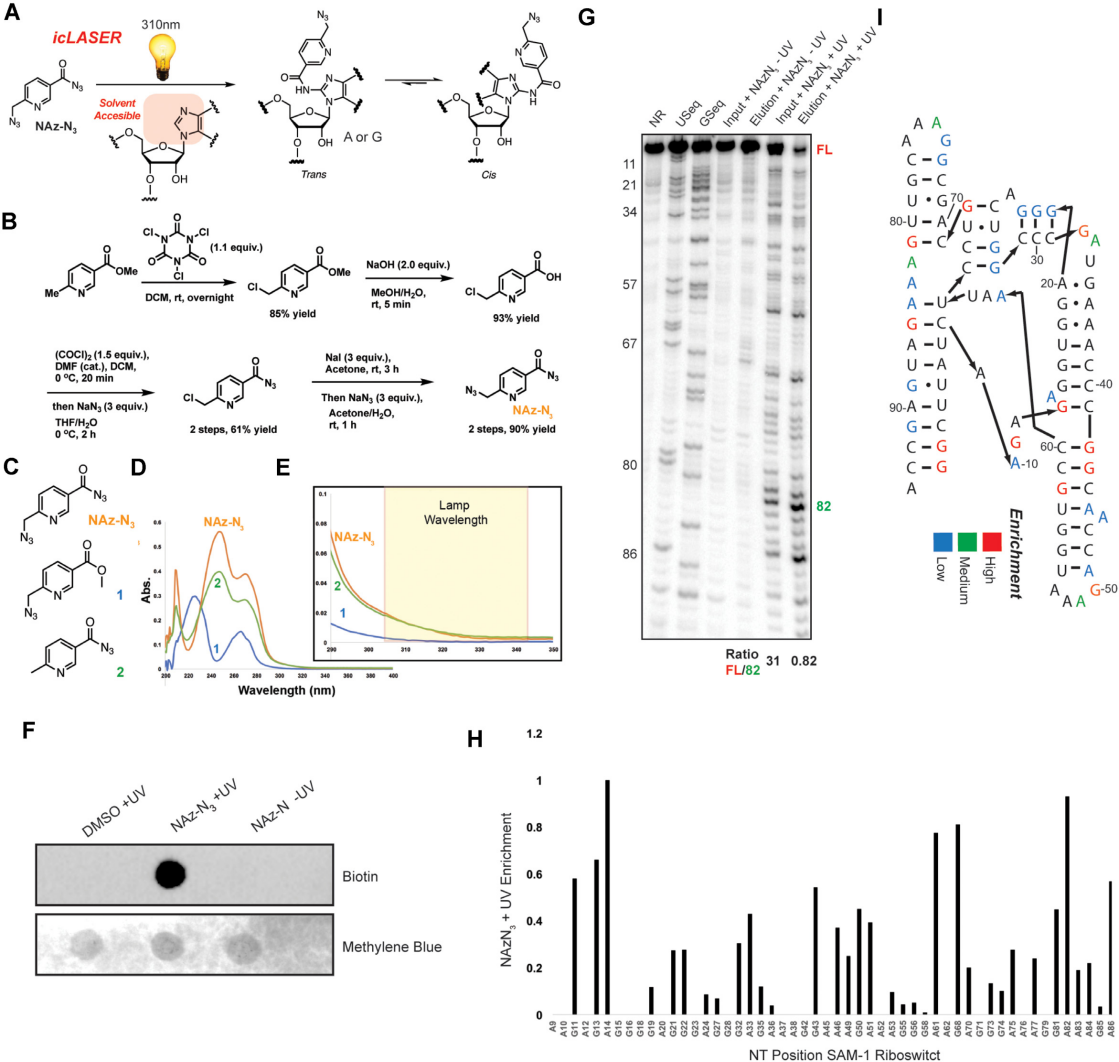
Development of a bi-functional probe for icLASER. (**A**) Reaction schematic for LASER, with C8 amidation utilizing NAz. (**B**) Synthetic scheme for the probe NAz-N_3_. (**C**) Structure of NAz-N_3_ and control probes for analysis of UV-VIS spectroscopy and light activation. (**D**) UV–VIS spectra of compounds from panel C. (**E**) Zoomed in UV-Vis spectra and corresponding wavelength used for light-activation of NAz-N_3_ and control compounds in panel C. (**F**) Streptavin-HRP dot blot of NAz-N_3_-modified RNA. RNA was incubated with NAz-N_3_ in the presence of long-wavelength UV light. RNA was precipitated and conjugated with biotin using SPAAC, as denoted in Figure 1. Following SPAAC, RNA was blotted. (**G**) Denaturing gel electrophoresis of modified SAM-I control RNA with NAz or NAz-N_3_. RNA was incubated with NAz-N_3_ in the presence of long-wavelength UV light. RNA was precipitated and conjugated with biotin using SPAAC. RNA was then enriched over magnetic streptavidin beads and eluted. Eluted RNA was reverse transcribed with ^32^P labeled primer and cDNA analyzed on denaturing gel. Ratio of full-length cDNA to modification was calculated against position 82 in the denaturing gel to demonstrate de-enrichment of the full length in comparison to the enriched modified position. (**H**) Integrated signal from panel G, for cDNA stops due to NAz-N_3_ modification of SAM-I RNA. NAz-N_3_. (**I**) Analysis of enriched positions from panel F.

Installing the enrichment moiety (i.e. alkyl azide) would optimally not reduce selectivity or efficiency of NAz in measuring nucleobase solvent accessibility. As such, NAz-N_3_, which we predicted would be able to undergo light transformation to the activated nitrenium ion, would also preserve the alkyl azide for Cu-free ‘click’ after RNA adduct formation. Notably, aroyl azides are amenable to activation by lower energy long wavelength light, whereas alkyl azides need higher energy short wavelength light for activation (20–22). We hypothesized these special characteristics of azide stability would permit photo-specific activation of NAz-N_3_ for icLASER probing (Figure 2A).

To test our hypothesis, we synthesized NAz-N_3_ and compounds **1** and **2** (Figure 2B; Supplementary Information). The NAz-N_3_ probe is synthesized efficiently from commercially available methyl 6-methylnicotinate in six steps in 43% overall yield. Treatment of methyl 6-methylnicotinate with trichloroisocyanuric acid generated monochloro substitution on the methyl group selectively, followed by alkaline hydrolysis of the ester to afford the corresponding carboxylic acid. The acetyl azide is installed routinely by transformation of the carboxylic acid into acetyl chloride using oxalyl chloride and azide substitution. The above monochloro substituent is finally converted to iodide through a Finkelstein reaction, which is replaced subsequently by azide to furnish NAz-N_3_. Synthesis of **1** and **2** is reported in Supplementary Information.

We obtained UV–Vis spectra of these compounds, to understand differences in their absorbance properties (Figure 2C), and observed that only NAz-N_3_ and **2** have absorption in the long wavelength UV region used previously in LASER and known to be specific for aroyl azides (Figure 2D and E) (14). Consistent with what others have observed with azide-specific activation, these data suggest that light activation of NAz-N_3_ should be specific to the aroyl azide.

We tested if NAz and NAz-N_3_ have similar chemical reactivity towards RNA (Supplementary Figure S1). We exposed the SAM-I riboswitch RNA to long UV light in the presence of NAz or NAz-N_3._ Reverse transcription with a ^32^P radiolabeled cDNA primer followed by denaturing gel electrophoresis revealed cDNA truncations at positions that matched NAz probing previously, in which NAz probing was demonstrated to be correlated with solvent accessibility of purine C8 positions (14). The *R*^2^ value between the two probes for cDNA truncations is 0.97. These results suggest that NAz and NAz-N_3_ have similar chemical reactivity towards a folded RNA.

We next sought to test if the DBCO conjugated NAz probe was amenable to enrichment followed by reverse transcription. We incubated the SAM-I riboswitch with NAz-N_3_ and exposed the solution to (+/−) long wavelength UV light. Following irradiation, we isolated the RNA and incubated the RNA with DBCO-biotin. We observed biotin conjugation on a streptavidin dot blot only in the sample incubated with long wavelength UV light and NAz-N_3_ (Figure 2F).

We next tested if NAz-N_3_ enriched RNA would produce similar truncation profiles to NAz probing (LASER). We used NAz-N_3_ modified riboswitch RNA, biotinylated the RNA using SPAAC, and subjected it to streptavidin-coated magnetic bead enrichment. Biotinylated RNA was then eluted and compared against input RNA. Each sample was then reverse transcribed with a ^32^P-cDNA primer and analyzed by denaturing gel electrophoresis. As shown in Figure 2G, the NAz-N_3_ enriched samples displayed significantly higher signal for structure stop cDNA and dramatically reduced full-length extensions. Finally, NAz-N_3_ elution mapped onto sites of modification similar observed with NAz (A and G residues; Figure 2H and I). We expect icLASER to enrich for RT-truncations and remove full length unmodified cDNA molecules. We integrated and compared the full length cDNA band (FL; Figure 2G), compared it to an arbitrary RT-stop (position 82), and calculated the ratio between the two bands. For input, this ratio was 31 and for enriched and eluted cDNAs this ratio was 0.82, demonstrating the power of enrichment and RT from enriched modified RNAs. Further, we also demonstrated that NAz-N_3_ can modify RNA and measure RNA structure in cells, and observed differences *in vitro* and in cells that correspond to cDNA profiles to that of the parent compound NAz (23) (Supplementary Figure S2). Overall, this result demonstrates that NAz-N_3_ is capable of enrichment using SPAAC reactions, yet still yields consistent truncation signatures for structural analysis.

### Transcriptome-wide implementation of NAz-N_3_

We modified K562 cells (*in vivo*) and K562 total RNA isolated and re-folded outside of cells (*in vitro*) in independent experiments with NAz-N_3_ (icLASER) and NAI-N_3_ (icSHAPE). We applied our well-established protocol for the isolation of poly-adenylated (polyA) RNA and mapping of biotin-conjugated RNA structure probes through deep sequencing (Figure 1, Materials and Methods) (16,24,25), which generated more than 100 million reads for analysis in each dataset (Supplementary Figure S3).

Mapping RT stops from icLASER enriched libraries revealed a marked enrichment of A and G residues, which was expected given the reactivity profile of the NAz-N_3_ probe (Supplementary Figure S3). icSHAPE enriched libraries had slight enrichment for adenosine and uridine residues, which is consistent with what we and others have observed (Supplementary Figure S3) (15,26). Importantly, we find a strong correlation between + UV samples, whereas the distribution of +UV versus –UV RT-stops showed very low correlation, suggesting RT-stops analyzed in the icLASER libraries are dependent on NAz-N_3_ an UV activation (Supplementary Figure S3). Transcriptome-wide libraries for biological replicates of icSHAPE and icLASER RT-stops showed strong intra-reagent correlation, comparable to other RNA structure probing efforts (26–28) and suggest that that each reagent measured a discrete set of positions in the transcriptome.

The interpretation of icLASER and icSHAPE probing on RNA should be viewed through the context of chemical reactivity. If the functional group is in the right environment to promote chemical reactivity, the electrophiles will form an adduct: a flexible 2′-OH reacts with the acyl imidazole of NAI-N_3_ and a solvent exposed C8 of purines reacts with the nitrenium ion of NAz-N_3_. We interpret both icSHAPE and icLASER RT stops as a residue that is flexible and solvent exposed, which often occurs in loop regions of RNA structural elements. A site that is not reactive to icSHAPE, but is reactive to icLASER, is a site that is not flexible, but is solvent exposed. Correspondingly, a site that is reactive to icSHAPE, but not icLASER, is a site that is flexible, but not solvent exposed. We have observed many sites that are reactive to both SHAPE and LASER electrophiles as well as those that have orthogonal reactivity; these reactivities are controlled by the local environment of the nucleobase (14,29,30). Comparison of reactivities from the same RNA pool have not been demonstrated and that was the main goal of our work described herein.

We first aimed to demonstrate that manual footprinting results were similar to those observed by deep sequencing. We analyzed the icLASER and icSHAPE data against manual footprinting on an RNA structure which had been characterized with LASER and SHAPE reagents: the ribosome. We focused on a section of 18S rRNA has been previously interrogated by footprinting (14) (Supplementary Note 1). The manual footprinting for NAI-N_3_ in this region showed some slight differences between in and outside of cells. Analysis of the RNA in this region revealed that it was solvent protected by rRNA and ribosome binding proteins, but was largely still single stranded and thus would be reactive toward SHAPE reagents. We also probed this region using NAz-N_3_ and observed marked differences between in and outside the cells. Consistent with this analysis was the reactivity profile for icLASER, which is supported by the solvent protected nature of this stretch of RNA in the 18S ribosome cryo-EM model. Overall, these results suggest that icLASER and icSHAPE are both measuring environment-unique aspects of RNA structure in and outside cells.

**Figure 3.**
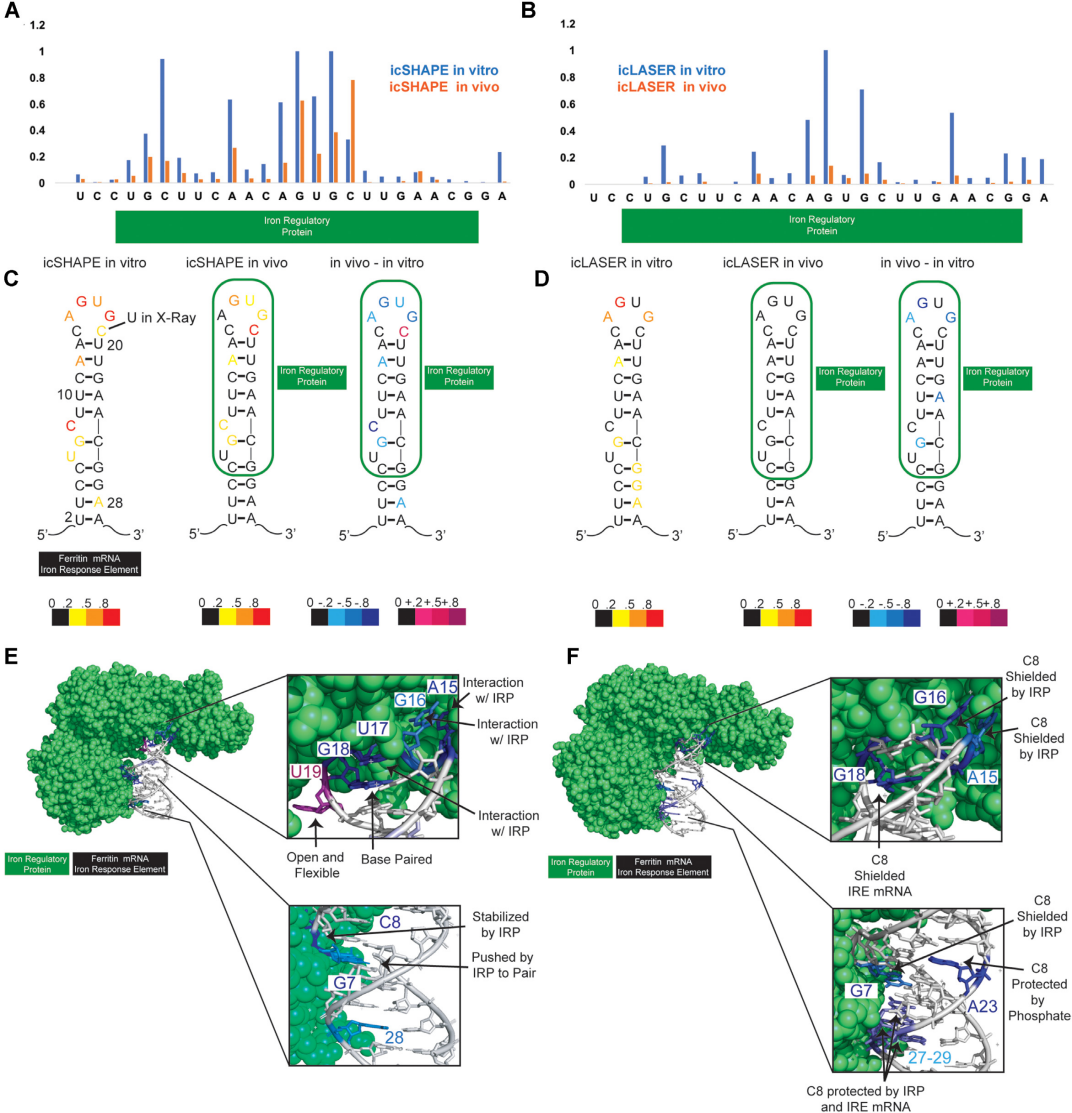
Analysis of icLASER and icSHAPE RT-stops. (**A**) icSHAPE profile at the IRE associated with heavy chain ferritin. The IRE genomic coordinates are chromosome seven from position 45459777 to 45459811. (**B**) icLASER profile at the IRE associated with heavy chain ferritin. (**C**) Secondary structure of the IRE element with icSHAPE data overlaid. (**D**) Secondary structure of the IRE element with icLASER data overlaid. (**E**) PDB model of the IRE/IRP complex with differential icSHAPE reactivities overlaid. (**F**) PDB model of the IRE/IRP complex with differential icLASER reactivities overlaid. PDB ID for the IRE/IRP model is 3SNP.

Beyond abundant transcripts like rRNA, we next examined a well-studied, but lower copy mRNA functional element via icLASER and icSHAPE, from the transcriptome-wide dataset. We focused on the iron response element (IRE) present in the untranslated region of some messenger RNAs and which binds proteins, modulating the flexibility and solvent accessibility of its nucleotides (31,32). In K562 cells, the IRE element within the Ferritin mRNA is bound by the iron response protein, controlling Ferritin gene expression (33). We compared *in vitro* to *in vivo* probing data on the ferrtin heavy chain mRNA with the expectation that the *in vitro* probing would display reactivity on the IRE RNA structure, and changes in the IRE reactivity profile would be observed *in vivo* due to protein binding. We observed differences at the IRP protein binding site in both icLASER and icSHAPE profiles (Figure 3A and B). Consistent with their chemical properties, icSHAPE RT stops fell on predicted single stranded regions and icLASER on A and G residues, regardless of their base-paired status, consistent with previous LASER probing experiments (Figure 3C and D) (14,30).

To gain a three-dimensional view of insights from the icSHAPE and icLASER data, we inspected of the co-crystal structure of the ferritin IRE and IRP (34) (Figure 3E and F). For icSHAPE, differences were observed in the stem and stem loop region. Nucleotides A15–U19 are all in regions in which the IRE-IRP interface, with A15-G18 making contacts with amino acids of the protein for main recognition. Key recognition of U17 is controlled by interactions with R269 and E302. G16 and A15 are interacting and likely being stabilized through extensive contacts with L551 and L262, respectively. These interactions cause U19 to become flipped out and limit its contacts, thereby likely making it more flexible and thus more reactive with the icSHAPE electrophile. Similar changes in reactivity at the IRE loop have been observed with purified RNA-protein complexes, where probe reactivity is significantly changed due to IRP binding (35). In the stem region, residues Guanine 7 and Cytidine 8, which are part of a kink in the helix, are likely to be flexible when outside of cells (no protein), and are forced into a more stable confirmation when protein is bound (in cells); these differences result in the observed lowered icSHAPE reactivity in cells. Cytidine 8 is a highly conserved flexible residue in the primary sequence of the IRE, and has been demonstrated to be important for enabling altered IRE RNA structure as a result of IRP binding (36). Cytidine 8 is protected and cupped through extensive interactions with amino acids R780, S681, P682, D781, W782 and R713 to stabilize its interaction with IRP. icLASER reactivities mapped to G and A residues, and inspection of the co-crystal structure in this context also revealed reasonable explanations for changes in reactivity in and outside of cells. Again, the loop region, notably residues A14, G15, G17 all had reduced reactivity and these residues have their C8 purine functional group protected by parts of the IRP protein and/or interactions with the IRE mRNA structure itself (Figure 3F). Amino acids L551, S371, K379, L262 and N298 provide a full shield of purine solvent accessibility. Positions 27–29 which have purine icLASER reactivity have decreased activity in cells, likely due to IRP interactions through amino acids 705–717 which coat the outside of the helix preventing NAz-N_3_ reactivity. Overall, these observations suggest that both icLASER and icSHAPE read out known structure motifs by measuring different chemical reactivities of RNA in complex pools of RNAs.

To fully characterize the icLASER dataset, a first-in-class transcriptome-wide map of solvent accessibility in human cells, we started by analyzing reactivity across mRNA functional elements. We observed that start codons are largely open and solvent accessible with high reactivity at the A and G of the AUG (Supplementary Figure S4). The stop codon also displayed high reactivity in the last two positions (Supplementary Figure S4), and both of these observations are in line with previous measurements using icSHAPE and DMS-seq (37). Next, we wanted to understand regions outside of canonical translational regulation that are regulated by contexts specific to living cells. To address this we compared the in vivo and in vitro profiles as previously described for icSHAPE (VTD = *in vivo* profile – *in vitro* profile, (15)). Specifically, we examined all hexamers across the transcriptome, and consistent with our previous reports, icSHAPE structure was overall higher *in vivo* (Supplementary Figure S5.). In contrast, we observed a bimodal distribution of VTDs due to solvent accessibility differences. Hexamers that include the Kozak sequence showed small VTD, which is in line with previous reports with icSHAPE (15). However, for icLASER, hexamers containing RNA binding protein motifs had lower maximal averaged VTD values, which can result in a change in chemical reactivity in cells due to protein footprinting (23,30), suggesting that icLASER could be used to map protein-RNA interactions. Overall, these results demonstrate the robustness of icLASER and icSHAPE probing and demonstrate their ability to define elements across the transcriptome that are potentially regulated inside living cells.

### Global prediction of RBP footprints by combining structure probing tools

Despite the importance of RNA-protein interactions and the interplay of RNA structure and protein binding, there has thus far been a lack of transcriptome-wide analysis of RNA structure motifs with the express goal of attempting to predict binding sites. Protein-centric techniques such as crosslinking and immunopurification (CLIP) are high resolution at the site of interaction but must be done one RBP at a time and do not directly report on the structural features of their binding sites (38,39). Conversely, RNA-centric footprinting methods (manual or transcriptome-wide) which could predict many RBP binding sites, have primarily focused on comparing *in vivo* versus *in vitro* conditions to assess protein binding, however this requires generating an additional *in vitro* dataset. Therefore, a simplified experimental and computational workflow that could robustly predict true RBP binding sites could offer substantial advantages to existing strategies.

**Figure 4.**
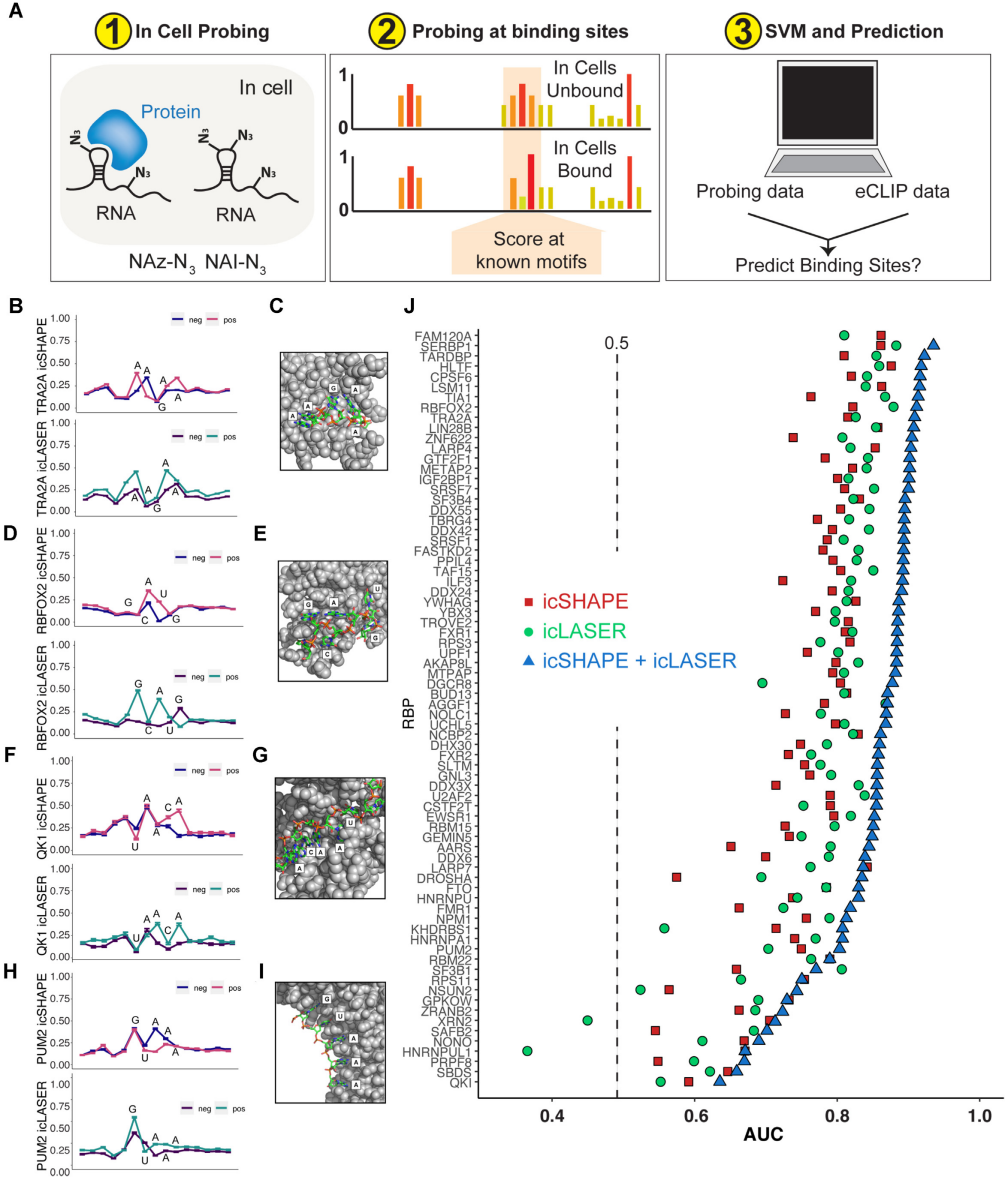
Utilizing icSHAPE and icLASER data to predict RNA-protein interactions transcriptome-wide. (**A**) Overview of methods to probe RNA structure in cells and test RNA-protein interactions in cells and predict binding using SVM models. (**B**) icSHAPE and icLASER difference maps between RNA probed inside living cells and in vitro centered at the motif for TRA2A. (**C**) X-Ray structure of TRA2A bound to RNA (PDB 2KXN). (**D**) icSHAPE and icLASER difference maps between RNA probed inside living cells and in vitro centered at the motif for RBFOX2. (**E**) X-ray structure of RBFOX2 bound to RNA (PDB 2ERR). (**E**) X-ray structure of RBFOX2 bound to RNA (PDB 2ERR). (**F**). icSHAPE and icLASER difference maps between RNA probed inside living cells and in vitro centered at the motif for QK1. (**G**) X-ray structure of QK1 bound to RNA (PDB 4JVH). (**H**) icSHAPE and icLASER difference maps between RNA probed inside living cells and in vitro centered at the motif for PUM2. (**I**). X-Ray structure of PUM2 bound to RNA (PDB 3Q0Q). (**J**) ROC analysis for predicting RNA–protein interactions using icLASER and/or icSHAPE structure probing. For each RNA-binding protein, we selected eCLIP bound sites *in vivo* and *in vitro*. A portion of this dataset was used as a training set, and the remainder was used to test the classifier. The classifier was trained using icSHAPE profiles, icLASER profiles, or both.

Given the orthogonality of icLASER and icSHAPE, we hypothesized that integrating their reactivity profiles simply from an in vivo experiment could provide a robust strategy to predict RNA-protein interactions (Figure 4A). We used enhanced CLIP (eCLIP) datasets from ENCODE collected from K562 cells as experimentally derived ‘true RBP binding’ sites (40–42). We focused on RBPs that bind in 3′-UTRs, as icSHAPE and icLASER probing performed on polyA RNA, we wanted to ensure high coverage in mature mRNAs. The selected RBPs had binding motifs with at least three A or G residues (resulting in 75 RBPs from the eCLIP database) and the mRNA targets were expressed at greater than or equal to 5 RPKM. True positive sites (+) were chosen as those with eCLIP data for a given RNA binding protein that has occupancy at a motif. True negative sites (−) were chosen as the same number of sites as the true positive, with the same motif, without evidence of occupancy by eCLIP.

To develop initial conceptual rules for this strategy, we started with focused analysis on four RBPs with published structural data (X-ray or NMR) and selected TRA2A, RBFOX2, QKI and PUM2. TRA2A (Figure 4B and C) is an RNA binding protein involved in the regulation of RNA stability and the formation of RNA granules (43). Analysis of structure probing at (+) and (−) sites revealed differences in icSHAPE and icLASER chemical reactivity towards the RNA. The 5′-adenosine is solvent exposed and makes limited contacts with the TRA2A protein: this may explain the higher icSHAPE reactivity at this position in the (+) sites. The next three nucleotides make substantial contacts with TRA2A (residues R188, P123, I195 and R111) that are likely stabilizing them and thus keep their icSHAPE reactivity low. The last adenosine residue is making contact with TRA2A (H-bonding with Y165), but this single H-bond may not be enough to fully stabilize that position and it might be dynamic in the interaction. icLASER shows strong reactivity at the 5′-adenosine, which is not paired with the protein and is solvent exposed. Interactions with the protein shield the C8 positions of the next A and G residues, which is consistent with the PDB model: these residues are protected by backbone phosphates, which are restricted by TRA2A binding. We have observed previously that when C8 positions of A and G are pushed up against the backbone phosphates of a given RNA, they are not reactive with LASER probes (23,29,30). The second pair of adenosines set is solvent exposed in the PDB model and these residues are also more reactive in the (+) sites.

RBFOX2 is an RBP that plays important roles in regulating RNA stability through interactions in the 3′-UTRs of mRNAs (44). RBFOX2 (Figure 4D and E) displayed a similar icSHAPE structure profile to our previous efforts with icSHAPE in mouse embryonic stem cells (15). Comparison of icSHAPE at (+) and (−) in vivo sites showed higher reactivity of the middle A and U residues. In the PDB model, these two positions are less interactive with the RBFOX2 protein. The first A is weakly H-bonded to the sugar face of the 5′-guanosine, with no contacts to RBFOX. The uridine oxygen-6 position is making a single hydrogen bond with B172 on the outside of the RBFOX structure. These interactions may be increasing the dynamic nature of these positions, in contrast to other positions in the motif which have tighter interactions with the protein. The icLASER signal of the first A and G exhibit higher reactivity at (+) sites, the last G is more protected by protein interactions and thus less reactive.

Quaking1 (QK1) is an RNA binding protein that is important for regulating mRNA trafficking and stability (45). (Figure 4F and G) icSHAPE reactivity showed nominal differences for three of the five positions. The first three UAA positions are making strong H-bonding and stacking interactions with residues G193, R130 and L103 respectively. Notably, the last C and A residues were more reactive, and these positions are not interacting with the QK1 protein. These positions also have the highest B-factors (thermal motion in the crystal) of all residues in the PDB model. For icLASER, the third A is more reactive at (+) positions and this adenosine, and in the model this A has its N7-C8 bond solvent exposed. The last A is both flexible and solvent exposed and as such has higher icLASER reactivity that sets the chemical environment of these purines for increased reactivity in icLASER.

Lastly, we inspected sites for the RNA binding protein PUM2, which is important in regulating RNA localization and translation (Figure 4H) (46). Comparative icLASER and icSHAPE profiles at in-cell eCLIP-annotated bound and unbound sites, yielded key differences that likely reflect protein binding (Figure 4I). For icSHAPE, the main differences were observed at the middle and second to last A positions, which in the model are base stacking with Y924 and H852. Despite these interactions the atoms in the third A have the highest B-factor value (>50 Å^2^), which is related to SHAPE reactivity (47). icLASER probing shows differences at (+) sites at the first G and third A positions: each of these has their C8 atom pointing away from the protein. The G and two A positions are in Watson-Crick-Franklin H-bonding interactions with the protein residues E963 and G891, respectively.

The four examples detailed above suggest to that differences in icSHAPE and icLASER signal, at the same primary sequence motif, differ in a manner related to their being bound by RBPs in living cells (eCLIP bound vs not bound). To generalize this observation, we implemented a Support Vector Machine (SVM) algorithm to learn icSHAPE and icLASER signals in an effort to predict sites of RBP binding. We evaluated performance of the SVM using the area under the receiver operating characteristic (ROC) curve for each protein (Figure 4j). Using this approach, icLASER or icSHAPE data were independently able to predict between 50–70% of the binding sites for 75 RBPs; quantitatively, the prediction was on average higher for icLASER than icSHAPE. However, when combined, icLASER and icSHAPE reactivities strongly improve the predictive power for every protein binding sites and were now able to predict at levels above 90% protein occupancy on RNAs.

Overall, these results support the notions that: (i) RNA solvent accessibility (icLASER) and RNA flexibility (icSHAPE) probing can be utilized to predict RNA-protein interactions, (ii) icLASER and icSHAPE data can be utilized to complement CLIP datasets to further support protein occupancies determined by orthogonal methods and (iii) by combining icLASER and icSHAPE, robust and transcriptome-wide predictions of many RBPs is possible without protein-centric techniques such as CLIP. We suspect the robust, but in some instances imperfect prediction of RBP binding sites may be due to the inability of icLASER and icSHAPE probing to differentiate changes in (A) protein binding that alter icSHAPE and icLASER reactivity from (B) changes in RNA structure that cannot be accounted for in all instances. Despite this, our SVM was trained only on 8 physical experiments (2x icSHAPE + unmodified, 2× icLASER + unmodified) while the data for the 75 eCLIP profiles resulted from over 300 individual experiments. Thus, chemical probing of RNA reactivities offers a simplified and multi-RBP approach to define binding sites in living cells.

### Predicting polyA sites in RNA with structure probing

**Figure 5.**
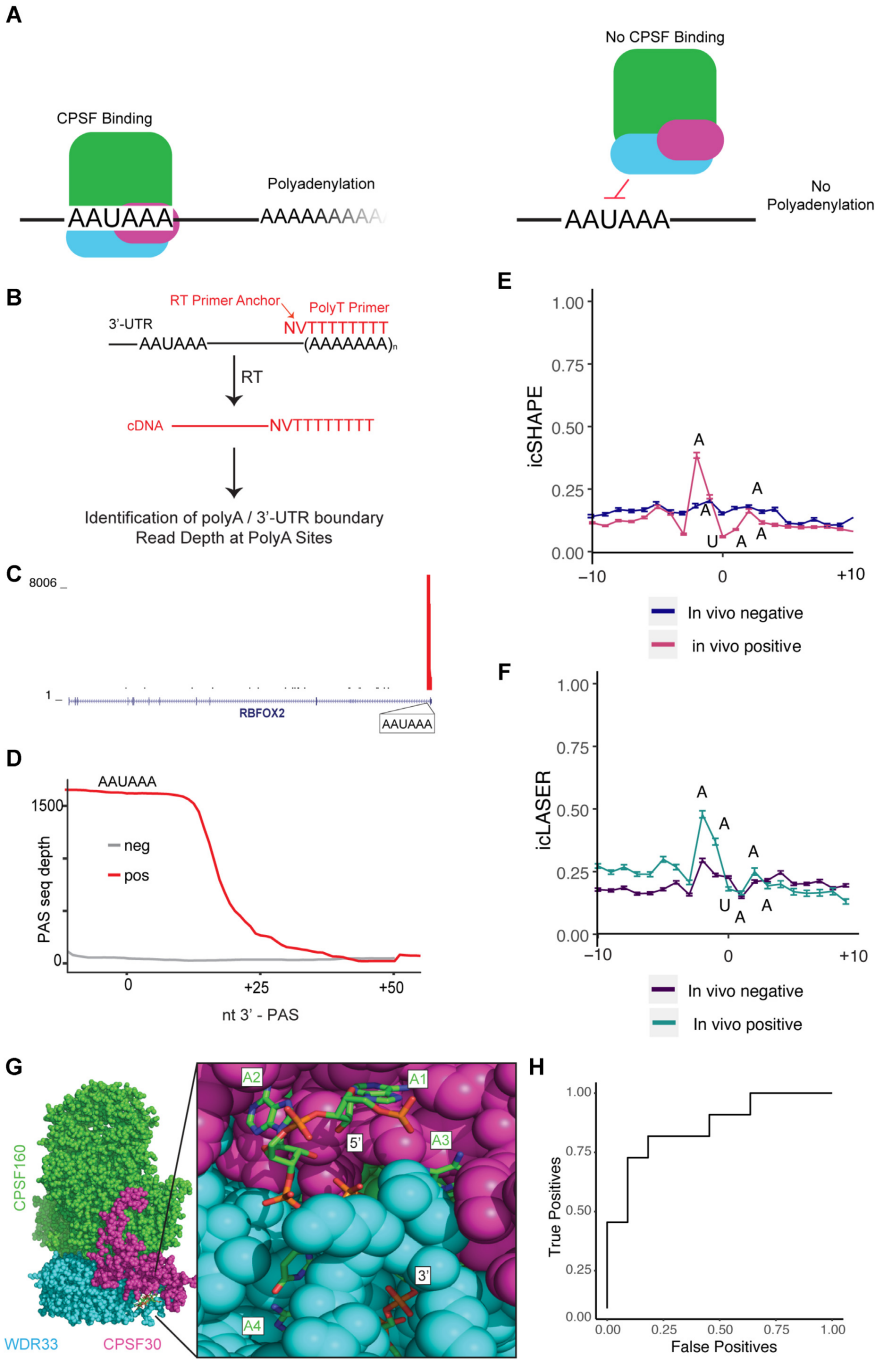
Utilizing icSHAPE and icLASER data to predict RNA polyA sites transcriptome-wide. (**A**) Figure illustrating the binding of CPSF complex to the PAS sequence, which results in eventual RNA processing and polyadenylation. (**B**) Schematic of polyadenylation sequencing (PASseq). (**C**) Genome browser track showing read density near the 3′-end of an RNA (RBFOX2) and demonstrating PAS seq specificity. (**D**) Cumulative read density at PASseq-determined *in vivo* and *in vitro* polyA sites. The zero point on the X-axis is centered on the uridine residue of the AAUAAA motif. (**E**) icSHAPE data comparing *in vivo* and *in vitro* RNA structure profiling at the PAS site. (**F**). icLASER data comparing in vivo and in vitro RNA structure profiling at the PAS site. (**G**) Structure of the cryo-electron microscopy structure of a quaternary complex of human CPSF-160, WDR33, CPSF-30 and an AAUAAA RNA (PDB 6BLL). (**H**) ROC analysis for predicting polyA sites using structure probing. The classifier was trained using icSHAPE and icLASER profiles.

Having demonstrated the utility of combining icLASER and icSHAPE reactivities in predicting actively bound RBP sites across the transcriptome, we text if this strategy could similarly predict another class of RNA functional elements. One important set of elements are the polyadenylation signals (PAS) that is controlled through protein recognition of a specific motif, resulting in cleavage and polyadenylation (48,49). Nearly all eukaryotic messenger RNA precursors must undergo cleavage and polyadenylation at their 3′-end for maturation. A crucial step in this process is the recognition of the PAS, 5′-AAUAAA-3′ by the CPSF complex (50) (Figure 5A). We next sought to determine if structure probing alone could be used to footprint the CPSF binding and eventual RNA processing and polyadenylation.

To experimentally determine PAS sites, for SVM analysis, we generated the first polyadenylation sequencing data (PAS-seq (51)) for K562 cells. PAS-seq uses polyA tail priming to identify the sites of polyA tail selection (Figure 5B). Inspection of PAS reads demonstrated clear read buildup at the 3′-end of transcripts (Figure 5C) and we obtained sites of high and low PAS read depth, corresponding to active or inactive elements, respectively (Figure 5C). We then compared the icLASER and icSHAPE profiles at the PAS sites and sequences with the same motif which are not annotated to be PAS sequences (negative control) (52). We noticed a striking structural difference between them: a large peak at the first two adenosine residues, followed by a drop in icLASER signal for the remaining UAAA (Figure 5D and E). To understand if these differences were related to a biophysical conformation of the RNA in an active PAS-recognition complex, we examined a newly published structure (53). This structure contains three large proteins which form into a complex and recognize the AAUAAA motif (Figure 5E). Close inspection of the structure revealed that the two five prime adenosine residues are completely solvent exposed, whereas the UAAA residues are solvent protected by protein binding. Impressively, the two AA residues have their C8 positions (site of icLASER reaction) completely exposed (Figure 5E). Consistent with our icLASER data, sites with high icLASER signal at the first two adenosine residues had very high coverage by PAS-seq. This data further suggested to us that icLASER signal alone could be used to predict polyA site selection. To test this hypothesis, we utilized SVM and demonstrated that icLASER and icSHAPE used together had an AUC of 0.87 for predicting PAS sites (Figure 5F). These data demonstrate that icLASER (and combined icLASER and icSHAPE) can be used to predict sites of posttranscriptional regulation and could be integrated with orthogonal datasets to interpret posttranscriptional processing of RNAs.

## DISCUSSION

Here, by developing novel RNA structure bi-functional probes, we extend the utility and flexibility of our previously reported RNA solvent accessibility probe, NAz. The new reagent, NAz-N_3_, similarly relies on specific photoactivation of an aroyl azide by long-wavelength UV light, but can subsequently be ligated to biotin using copper-free ‘click’ chemistry, for enrichment of modified sites of adduct formation. Using this chemistry, we measured RNA solvent accessibility in K562 cells, transcriptome-wide.

With the development of transcriptome-wide RNA structure probing techniques, an exciting but thus far poorly explored aspect of these data has been the possibility to infer interactions between RNAs and their cellular partners. By employing a computational strategy (SVM) to combine icSHAPE and icLASER (as well as other reactivity-based measurements) we take a critical step towards learning the potential of these methods in predicting RBP binding and other functional RNA processing activities like PAS selection. We demonstrate the power of this approach by predicting RNA-protein binding sites for a large number of RNA binding proteins. We demonstrate that such an approach could be very powerful for measuring RNA-protein interfaces and is highly complementary with protein-centric methods such as eCLIP. Further, we utilize RNA structure probing to identify a structure signature associated with polyadenylation sites, which is due to the presence of a protein bound at the PAS site. This extension also enables structure probing to be utilized for other aspects of RNA biology, such as RNA processing.

The complexity of nucleic acid polymer structure is now well appreciated for DNA and the folding of the genome; importantly, measuring the structure of such nucleic acids and how proteins interact with DNA has been incredibly valuable for understanding how the genome is regulated to control biological processes within cells. Our combined icSHAPE-icLASER strategy make headway toward this goal for RNA, by the continued development of tools for transcriptome-wide measurement of RNA structure and RNA interactions that can contribute to its biological function and regulation. We anticipate that icLASER (a new aspect of transcriptome-wide RNA structure probing via solvent accessibility) will become an increasingly useful chemical tool to probe RNA structure in living cells transcriptome-wide. By demonstrating the predictive power of these tools, we expect RNA structure probing to expand its value into aspects of RNA biology.

## DATA AVAILABILITY

icLASER and icSHAPE datasets are deposited on GEO under the accession number GSE132099.

Additionally, icSHAPE datasets are deposited on the ENCODE database under experiments ENCSR976RFC, ENCSR803XFA, ENCSR286LXS, ENCSR992XHC, ENCSR052BBY, and ENCSR836VQU.

PAS sequencing datasets are deposited on GEO under the accession number GSE145400.

## Supplementary Material

gkab885_Supplemental_Files
