# Supplementary material for: Diverse functional elements in RNA predicted transcriptome-wide by orthogonal RNA structure probing
Source: Nucleic Acids Res. 2021 Oct 11;49(20):11868–82. doi: 10.1093/nar/gkab885 (PMC8599799; doi:10.1093/nar/gkab885)
Supplement: gkab885_Supplemental_Files [file gkab885_supplemental_files.zip › 210803_NAzN3_Supplementary_Note1.docx]

Supplementary Note 1:

Discussion of icLASER and icSHAPE reactivity on ribosomal RNA.

Diverse functional elements in RNA predicted transcriptome-wide by orthogonal RNA structure probing

Dalen Chan^1,$^, Chao Feng^1,$^, Whitney England^1,$^, Dana Wyman^2^, Ryan A. Flynn^3^, Xiuye Wang^4^, Yongsheng Shi^4^, Ali Mortazavi^2^, and Robert C. Spitale^*,1,5.^

1. Department of Pharmaceutical Sciences, University of California, Irvine. Irvine, California. 92697, (2) Department of Developmental and Cellular Biology, University of California, Irvine. Irvine, California. 92697 (3) Department of Chemistry, Stanford University, Stanford CA 94305 (4) Department Microbiology and Molecular Genetics, University of California, Irvine. Irvine, California. 92697 (5) Department of Chemistry, University of California, Irvine. Irvine, California. 92697

($) These authors contributed equally to this manuscript.

*Correspondence: rspitale@uci.edu

To compare icLASER and icSHAPE with manual footprinting we focused on a highly abundant RNA, rRNA. We also took advantage of an available high-resolution cryo-EM structure that had been determined to (2.9 Å)[^1^](#_ENREF_1). This permits a molecular level analysis of LASER probing in comparison to structural states representative of the cellular environment.

We focused on residues within H15 and H16 as these residues have been observed by the Cryo-EM mode to be both single stranded and solvent protected. Further, in the secondary structure model of the 18S rRNA, these residues are predicted to be single stranded when the ribosome is not interacting with proteins in a re-folded state outside the cell[^2^](#_ENREF_2). As such, we believed these would be useful for comparing footpriting because they should result in very different chemical reactivity profiles. icSHAPE should have high reactivity inside and outside the cells. icLASER should have less reactivity inside cells in comparison to outside cells because it should be solvent protected in those regions. To test this, we performed icSHAPE and icLASER on both conditions.

In H15 and H16 the majority of the helices are hidden from solvent, due to extensive interactions with other sections of 18S rRNA and ribosomal proteins S9, S24, and S30. We have subjected this part of the ribosome to manual footprinting before against NAz and NAI, the parent molecules for the azide-derived NAI-N_3_ and NAz-N_3_ used herein[^3^](#_ENREF_3).

As expected, *In vitro* LASER modified many of the residues connecting H15 and H16, in the adenosine-rich sequence (**Figure 3, c-e**). However, in stark contrast, these residues did not show LASER reactivity in cells. These results for Naz-N_3_ are consistent with our previous reports with the parent molecule Naz (LASER)[^4^](#_ENREF_4). When mapping the interaction of these residues back onto the cryo-EM model, analysis revealed that many of them are in direct contact with the three rRNA binding proteins. The C-8 position of these residues are packed tightly against the structure of ribosomal protein S9 and preventing interaction with solvent (**Supplementary Note Figure 1**). We also benchmarked the icSHAPE probe (NAI-N_3_) against the parent molecule NAI and observed reactivity on many residues within H15 and H16. These residues are single stranded or are in non-optimal weak base pairs in the cryo-EM structure. Consistent with the known reactivity of SHAPE probes and the NAI nicotinoyl imidazole electrophiles, both NAI-functionalized probes were reactive with the known single stranded residues in the long single-stranded stretch of residues G513 and A526. As such, icLASER and icSHAPE probes have similar reactivity profiles as their parent molecules. Further sequencing-based approaches used herein have reasonable agreement between manual footprinting and sequencing.


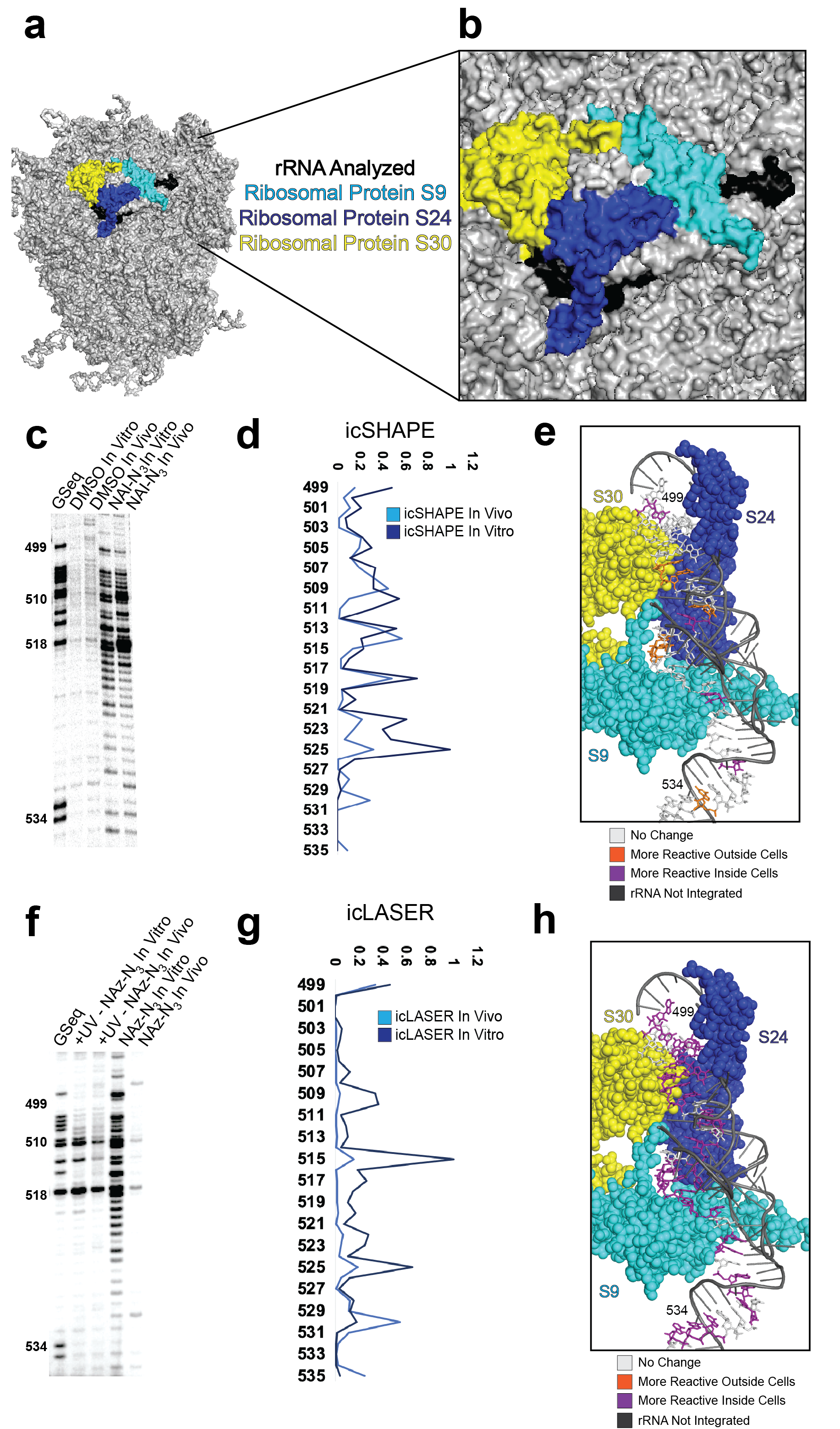


**Figure for Supplementary Note 1: Analysis of icLASER and icSHAPE RT-stops. a.** CryoEM model of the 80S ribosome (PDB 4v6x). **b.** Zoom in of rRNA section interrogated in for comparison footprinting. **c.** Denaturing gel analysis of NAI-N_3 ­_reactivity. In vitro re-folded RNA or cells were subjected to incubation with NAI-N_3_. Modification sites were analyzed by reverse transcription. **d.** icSHAPE reactivity profile over the same 18S rRNA region as in panel c. **e.** Structural analysis of 18S rRNA with differential reactivity from icSHAPE colored. **f.** Denaturing gel analysis of NAz-N_3 ­_reactivity. In vitro re-folded RNA or cells were subjected to incubation with NAz-N_3_. Modification sites were analyzed by reverse transcription. **g.** icLASER reactivity profile over the same 18S rRNA region as in panel f. **h.**  Structural analysis of 18SrRNA with differential reactivity from icLASER colored.

**References for Supplementary Note 1.**

1 Khatter, H., Myasnikov, A. G., Natchiar, S. K. & Klaholz, B. P. Structure of the human 80S ribosome. *Nature* **520**, 640-645, doi:10.1038/nature14427 (2015).

2 Petrov, A. S. *et al.* Secondary structures of rRNAs from all three domains of life. *PLoS One* **9**, e88222, doi:10.1371/journal.pone.0088222 (2014).

3 Feng, C. *et al.* Light-activated chemical probing of nucleobase solvent accessibility inside cells. *Nat Chem Biol* **14**, 325, doi:10.1038/nchembio0318-325 (2018).
